# Screening for frailty: older populations and older individuals

**DOI:** 10.1186/s40985-016-0021-8

**Published:** 2016-08-22

**Authors:** Brigitte Santos-Eggimann, Nicolas Sirven

**Affiliations:** 1 Institute of Social and Preventive Medicine, Lausanne University Hospital and Faculty of Biology and Medicine, Lausanne, Switzerland; 2 LIRAES, University of Paris Descartes, Sorbonne-Paris-Cité, 45 rue des Saints Pères, 75006 Paris, France

**Keywords:** Frail, Frailty, Screening, Public health, Clinical setting, Primary care, Hospital, Surgery

## Abstract

The concept of frailty as a health dimension in old age is recent and has its origin in the development of geriatric medicine. Initially an unformulated clinical intuition, it is now defined by a diminished physiological reserve of multiple organs that exposes older individuals to increased vulnerability to stressors and a higher risk of adverse outcomes.

The operational definition of frailty, however, is still debated. From a diversity of models, two emerged in the early 2000s from epidemiological studies conducted in large population-based aging cohorts. The body of research emphasized prospective associations between a frailty phenotype and a range of adverse outcomes or between a frailty index measuring the accumulation of deficits and death. A few studies showed promising spontaneous remissions in the early stages of frailty, raising expectations for effective interventions. Transitions between frailty stages and effective interventions on frailty nevertheless remain two fields needing further investigation.

More recently, these tools have been applied as screening instruments in clinical settings to guide individual decision-making and orient treatments. New questions are raised by the use of instruments developed to screen frailty in epidemiological research for assessing individual situations. Inquiring whether frailty screening is relevant opens a Pandora’s box of doubts and debates.

There are many reasons to screen for frailty both from a public health and a clinical perspective that are only exacerbated by the current demographic evolution. Open questions remain about the feasibility of frailty screening, the properties of screening tools, the relevance of an integration of socioeconomic dimensions into screening tools, and the effectiveness of interventions targeting frailty. Fifteen years after the publication of the Fried and Rockwood landmark papers proposing operational definitions of frailty, this article presents an overview of current perspectives and issues around frailty screening in populations and in individuals.

**Recommended citation:** Santos-Eggimann B, Sirven N. Screening for frailty: older populations and older individuals. *Public Health Reviews*. 2016.

## Introduction

In just 15 years, the concept of frailty in old age has significantly grown in importance, as evidenced by numerous research publications and its inclusion in most gerontological conference programs. Two current features of demographic aging explain our sustained interest in frailty despite a persistent lack of consensus about its operational definition and our limited knowledge about its causes.

First, increasing longevity gives chronic diseases the time to manifest, develop, and evolve, leading to consequences such as frailty and functional decline. Although centenarians were exceptional a few decades ago, they no longer are today. The burden of disability from advanced age is now highly visible and expected to increase with continuing progress in life expectancy. The fast growth in numbers in the oldest age category not only creates a need to develop long-term care services but also has a strong impact on all healthcare settings. Older patients represent a major share of primary care physician consultations, emergency room visits, and acute hospital admissions. Although disability is frequent in old age, health state is heterogeneous, and many older individuals are robust. As surgical procedures and invasive medical treatments are increasingly performed on geriatric patients, physicians are often confronted by the need to evaluate the frailty of an older patient.

Second, past demographic events, such as the post-World War II baby boom, will influence our near future. A large generation is currently reaching its third (active retirement) age, with the prospect of a high residual life expectancy. Chronic diseases are already present in this population at a clinical, and possibly subclinical, stage with a high prevalence. The second quarter of this century will witness the fourth (dependent) age of this numerically consequent cohort, with high risk of an epidemic of age-related disability and resulting pressure on healthcare systems as a whole. The coming years offer a window of opportunity for interventions to reduce the impact of this demographic event of the past on the health of aging populations, with frailty as a central target.

Although demographic circumstances justify investments in frailty research, the many unknowns around it raise questions about the pertinence and feasibility of screening for frailty in old age, from both public health and clinical perspectives. Basic conditions for screening are that we know what we are screening for (i.e., frailty can be defined); that without screening the characteristic we screen for would remain unobserved; that we have adequate instruments of detection that are sensitive, specific, and predictive; and that screening results can prompt effective intervention or management decisions. This article reviews the developments of research on frailty in older populations and individuals since the turn of this century speaking for or against screening for frailty in a context of scarce knowledge and urgency.

## Frailty at the turn of the twenty-first century

### Do we know what we want to screen for?

The concept of frailty is recent, as is geriatric medicine: it emerged during the last quarter of the twentieth century. The gap between chronological and biologic age of older individuals—and the need to consider the second rather than the first to treat older patients appropriately—is at the heart of clinical practice in geriatrics. Although one or more chronic conditions are in most cases present in older patients, at any given age, their number, combinations, severity, and impact on functional capacities are very diverse, so that although the prevalence of chronic diseases is highest in older age, the chronologic age of an individual does not necessarily equate with the risk of disability and death. The frailty of an older person is often expressed as a level of intrinsic vulnerability. It tends to increase with chronological age but it is not confounded with it.

Likewise, frailty may be the precursor of a progressive dependency in activities of daily living (ADL); therefore, many dependent older individuals are both frail and disabled. However, all disability in old age does not result from frailty, and all frail individuals are not necessarily functionally dependent. After some debate, frailty is now considered to be a distinct health dimension, aside from comorbidity and functional dependency, and as a pre-disability stage [1].

Although the definition of frailty is still under dispute, there is some consensus around considering it a health condition “with multiple causes and contributors that is characterized by diminished strength, endurance, and reduced physiologic function that increases an individual’s vulnerability for developing increased dependency and/or death” [2]. Whether or not frailty may exist in the absence of multiple chronic conditions and functional loss is nevertheless not yet established. First, chronic diseases may be present but still at a preclinical stage or undiagnosed in some frail individuals who decline in their functional capacities without having met the threshold that delineates the need for help [3]. Second, frailty might also exist in some cases in the total absence of chronic conditions and functional decline. Frailty is a progressive health characteristic with potentially preventable negative consequences. In principle, screening for frailty is thus desirable, provided that we develop adequate instruments to identify it, particularly before reaching a state of functional dependency, and know enough to act on it.

Frailty is here conceptualized as a dimension of health. Its definition, however, also refers to vulnerability, another concept needing clarification. According to Chambers [4], vulnerability is a broader concept than frailty. It includes both frailty (intrinsic health characteristic) and exposure to shocks and stress that may be external. Therefore, vulnerability has additional, environmental components (including socioeconomic circumstances) which themselves are recognized determinants of health (including, potentially, of frailty). Frail individuals are vulnerable; environmental factors are both likely to increase their level of frailty and to have an independent effect on their level of vulnerability.

### Do we know how to detect and measure frailty?

Many models, definitions, and instruments were proposed at the turn of this century to operationalize the definition of frailty and identify frail individuals [5, 6]. Two approaches emerged from prospective, quantitative research conducted on large samples of community-dwelling populations, both described in landmark papers published in 2001. The frailty phenotype defined by Fried et al. [7] refers to the multisystem loss of physiological reserve that delineates frailty as a risk for a range of adverse outcomes. Fried’s frailty phenotype relies on a biological conceptual model in which frailty has close links with sarcopenia, neuroendocrine decline, and immune dysfunction [8, 9]. It is based on the observation of five characteristics (shrinking, weakness, slowness, exhaustion, and low activity), each measured by one single criterion derived from a secondary analysis of data from the Cardiovascular Health Study (CHS). Shrinking is defined by weight loss, weakness by low grip strength measured with a dynamometer, slowness by low walking speed, exhaustion by self-reported fatigue based on two items from a questionnaire designed to assess depressive symptoms, and low activity from an estimate of energy expenditure based on a leisure-time activity questionnaire. All criteria are considered equally to classify individuals as frail (3 to 5 criteria), intermediate or pre-frail (1 or 2 criteria), or not frail (none). The large diffusion of Fried’s frailty phenotype may be due to its face validity and at least partly to the limited number of variables that need to be measured. It has nevertheless been criticized for reducing frailty to the physical aspects of health, thus neglecting mental health problems that are frequent in old age such as mood disorders or cognitive limitations that may contribute to frailty [3]. However, the five indicators proposed by Fried et al. are likely to reflect mental health as well: weight loss, fatigue and low physical activity are observed in depression and dementia; fatigue is measured by items from a depression screening tool; and recent research has pointed to the relationships between frailty and cognitive limitations [10, 11]. Furthermore, multiple correspondence analyses performed in several populations on the five phenotype dimensions with depression and cognition suggested that all belong to a common construct [12].

The “accumulation of deficits” model, described by Mitnitski and Rockwood, relies on a frailty index computed from a large number of health variables [13]. It refers to the concept of advanced biological age related to the risk for dying. This model was developed based on data from the Canadian Study of Health and Aging, making use of more than 90 individual variables such as medical diagnoses, self-reported health problems or symptoms, signs, results of laboratory tests, or functional difficulties in ADLs. Each variable contributes equally to the frailty index, which is defined as their arithmetic sum. Three principles guide the selection of variables: they indicate health problems of increasing prevalence with age, they point to more than one system, and they do not reflect conditions that are universally present in old age (and would thus not discriminate between individuals of the same chronological age). In theory, the set of variables selected to compute the index of frailty may change between different samples as long as the number of variables included is large, at least 30 to 40 [14]. The “accumulation of deficits” model does not provide clues to the underlying physiological mechanisms leading to frailty. However, the large range of health deficits measured to compute the frailty index takes into account the multidimensionality of frailty, including its physical and mental aspects. The frailty index has two other valuable characteristics: it can be computed from any database that provides a large set of health indicators, and it measures frailty on a continuous scale.

The frailty phenotype and the frailty index thus coexist as complementary tools [15] to detect frailty in large samples of the population. In a recent comparison of the two instruments, the frailty index identified a much higher proportion of the community-dwelling population as frail [16]. Other instruments, such as the Tilburg Frailty Indicator or the Groningen frailty indicator, are designed to capture frailty in old age as a more global concept and include psychosocial characteristics [17, 18]. By including psychosocial factors, these instruments tend towards the broader concept of vulnerability. When applied in the same population, they classify as frail an even larger proportion than the frailty index and, unfortunately, the currently available instruments designed to detect frailty poorly agree with each other [19].

All instruments used today to identify frailty were essentially validated through demonstration of their prospective association with adverse outcomes in population-based cohort studies. In general, the frailty index is more predictive of death than the frailty phenotype [16, 19]. However, its definition includes indicators of disability; as a consequence, it cannot be used to predict the incidence of functional decline. The frailty phenotype, in its original definition or in versions adapted to available data, has been found to be independently associated with the incidence and worsening of functional decline in several cohorts [7, 20–22]. Frailty may not only progress towards more severe stages [23] and lead to disability but it may also regress spontaneously, particularly early in its course, as shown in studies with measurements based either on the frailty phenotype [24–26] or on the frailty index. [27, 28] This natural history underlies the hope that a better understanding of frailty and its determinants will result in the design of effective interventions aiming at both a slower progression towards more severe stages and at less unfavorable functional outcomes.

## Frailty screening and public health in aging societies

Although the concept of frailty originated in the clinical concerns of geriatricians for their patients, it was essentially developed through population-based epidemiologic research and should have important applications for the practice of public health in aging societies. Monitoring population health, planning for appropriate health services, and identifying subgroups at higher risk for preventive actions to avoid adverse health outcomes are basic activities that potentially could benefit from screening for frailty in older general populations.

### Frailty and monitoring of older populations’ health

Because chronic conditions and their consequences are related to old age, a degradation of the population health is expected in most countries, for structural reasons, with the phenomenon of demographic aging. The extent to which younger cohorts, who benefit from a higher life expectancy, are in better health when they reach retirement age is unsure and appropriate indicators are needed to estimate the level of health, particularly in the “young-old” age category. The evaluation of population-based interventions and of changes in the organization of healthcare systems promoted under the auspices of health and aging policies should also rely on health indicators that are pertinent for all age subgroups of the older population.

The usual health indicators, like the frequency of specific diagnoses, are not appropriate for comparing the health of older populations over time, between places, or among subgroups of the population delineated by non-medical criteria. Socioeconomic characteristics may influence care-seeking behaviors and the access to diagnostic procedures for several reasons, including perceptual, cultural, and financial, leading to biased comparisons of morbidity. Chronic conditions need to be diagnosed before being reported, assuming that the individual did not postpone or forfeit healthcare some time before the survey interview. Furthermore, specific diagnoses are only partly relevant to describing health at an age of widespread comorbidity. Disability indicators, although appropriate for measuring health in older populations, have limitations because they are mostly relevant at the age of 80 and more. They are not so helpful in describing the health of older populations when a large proportion is in the third age, which will be the dominant situation in the coming decade. Screening for frailty provides a complementary, diagnosis-independent, indicator of health relevant to the whole range of old age and quantifying its vulnerability.

### Frailty as an indicator of need for health services

Planning for healthcare resources is another public health task for which assessing the level of frailty in the population may be useful. Both the frailty phenotype and the frailty index are associated with the use of health care in the community [16, 29]. The frailty phenotype is also associated with admission to nursing homes [20], and the Tilburg Frailty Indicator seems to be linked to the use of a wide range of health services [30]. By contrast, in severely disabled older persons living in the community, frailty was not associated with more frequent visits to emergency departments [31]. However, relationships between frailty and health services use in general community-dwelling populations deserve more research because several hypotheses could explain them. Frailty might result from treatments that imply multiple contacts with health services either for prescription or for the treatment of secondary effects. It could also be the consequence of inappropriate incentives for healthcare systems, like premature hospital discharge of frail patients in diagnosis-related group payment systems or the neglect of secondary or tertiary prevention in time-rationed consultations, resulting in health decompensation and the need for costly care. A perception of frailty by older patients in the absence of detected disease or functional loss may also lead to a feeling of unmet need and multiple demands on health services.

### Frailty as a target of population-based interventions and public policies

The third reason to screen for frailty in older populations is the possibility of designing and implementing preventive, population-based interventions targeting identified risk factors. Achieving this aim will necessitate more investment both in epidemiological research on the determinants of frailty and its consequences, with an emphasis on modifiable risk factors for frailty incidence, worsening and evolution towards disability, and in the evaluation of the effectiveness of interventions in various populations and their subgroups. Although much effort has been invested in the definition and validation of screening instruments, we still know little about these two aspects besides repeated observations of higher levels of frailty in women [7, 32] and in socioeconomically disadvantaged subgroups [33–36]. Therefore, frailty screening instruments used to identify subgroups at risk should be limited to health dimensions and keep socioeconomic characteristics apart, as potential explanatory risk factors for frailty on which non-medical action could be taken. Socioeconomic circumstances also remain to be investigated as independent components of vulnerability that may interact with frailty as determinants of adverse health outcomes.

If early stages of frailty are the most appropriate target for intervention—because they are more likely to be reversible—and if they correspond to preclinical (or undiagnosed) chronic conditions and functional loss, a first modifiable factor is the capacity of medical care to identify hidden problems. Healthcare systems were designed in the past century mainly to respond to the acute care needs of a young population. Their initial response to population aging was an increase in nursing home supply. It was followed by the development of home care and help services, supported by intermediate care services such as respite care or sheltered housing and, more recently, by efforts to coordinate the care provided by a growing diversity of services. It is unclear, however, that this re-organization of health services succeeded in encouraging health professionals, through adequate financial incentives, to invest their time and resources in the detection and systematic treatment of hidden chronic diseases, dental problems, sensory deficits, functional losses, mood disorders, and cognitive impairments, all factors that most probably signal the incidence of frailty and influence its course. Adaptations of health insurance and social security policies are primary targets to correct these frequent and sometime neglected health problems in old age. Socioeconomically disadvantaged populations may be particularly sensitive to this type of intervention. The French experience of social policy agencies, currently screening retirees for frailty to offer targeted reinforced care and support to vulnerable individuals, is an example of a promising public policy intervention [37]. It would require a detailed evaluation to demonstrate its effectiveness.

The observation of associations between social isolation and frailty points to another potential for action. A low level of physical activity and reduced social contacts may be improved when streets and post offices offer comfortable seats that permit older persons with mobility limitations to engage in a walk outside their home. Programs to promote friendly cities for the older population are an opportunity to test the effect of this type of interventions on frailty in old age. Other interventions such as programs to increase older persons’ computer and digital literacy may also help to keep them integrated in social exchanges. Whether or not these interventions would prove efficacious in reducing the level of frailty in older populations is unknown. They should be evaluated by appropriate monitoring of frailty indicators in population-based surveys.

### Measurement tools to compare the level of frailty in populations

For purposes of epidemiologic comparisons, the frailty index suffers from its reliance on medical diagnoses that reflect not only the population health but also the performance of healthcare systems, which is likely to vary over time, across regions and among population subgroups. From this point of view, the frailty phenotype may be more appropriate for comparing populations with differential access to care. Geographic variations of the frailty phenotype in older populations have been shown in the Survey of Health, Aging and Retirement in Europe (SHARE) study [36]. However, two conditions are necessary for epidemiological comparisons that contrast regions or population subgroups using Fried’s phenotype as a screening tool: the operationalization of its five dimensions must be identical in the compared populations, and thresholds for defining low walking speed, grip strength, and physical activity must not be adapted to each country. The cut-offs empirically determined in the CHS for these three dimensions by Fried et al. were validated by associations of the frailty phenotype with adverse health outcomes, including death and disability [7]. They can be used in populations of similar structure. Remaining bias in population comparisons may nevertheless result from the self-assessment of fatigue as a measure of exhaustion in Fried’s phenotype. The effect of a country-specific response style on the assessment of subjective health has been demonstrated in the SHARE survey [38]. Another, more logistical concern is the necessity of direct observation of performance tests to assess this phenotype, which results in a sizable proportion of non-random missing data with a disproportionate mortality observed in incompletely evaluated subjects [19]. The conduct of performance tests also generates higher costs in large population surveys. Therefore, recent studies have tried to identify more simple instruments to measure frailty in the community, using the frailty phenotype, the frailty index, or a comprehensive geriatric assessment as a reference. These studies are based exclusively on self-reported information collected in postal questionnaires [39, 40], use shorter instruments [41], or limit the observation to a single dimension [41, 42]. In this last case, a slow walking speed is found to be a sensitive test for detecting frailty. Other studies evaluated the predictive capacity of selected performance tests for mortality [43] or incidence of disability [44] in samples of the community-dwelling population. For both outcomes, walking speed has been identified as a useful risk indicator, one also highlighted in a review of physical frailty indicators predicting difficulties in ADLs [45].

## From the population to the individual

Whether patients should be screened for frailty in clinical settings is a more recent and controversial question. Several recommendations issued by agencies or after consensus conferences push for the application of frailty screening in patients [1, 2, 46, 47]. The most evident reason is the potential for prevention suggested by a spontaneous reversibility of frailty in non-disabled individuals in the early stages of frailty. This potential is nevertheless challenged by the current lack of specific interventions of proven efficacy and effectiveness for frail individuals [48].

Other motivations may justify the adoption of frailty screening in clinical settings. They result from the availability of invasive medical and surgical treatments that can benefit older patients even at an advanced age but can be deleterious in frail individuals. Some of these treatments are expensive, per se or as a result of their complications in frail patients. Screening for frailty is considered appropriate for discussing the risk of treatments with patients, guiding decisions between different care options, and reinforcing perioperative care when invasive treatments are necessary [49]. It has also been advocated to protect patients against age-based rationing of care as well as physicians against accusations of ageism [50, 51].

### Screening for frailty in primary care

A central question is the extent to which frailty screening instruments validated in population-based studies and showing prospective associations with adverse outcomes can accurately predict these outcomes at an individual level. Frailty screening instruments used to decide treatments in clinical practice must be sensitive so as to detect most patients needing special attention and specific so as to avoid denying effective treatments to robust patients falsely classified as pre-frail or frail. Available instruments tend to have high sensitivity but limited specificity. Frailty screening instruments must also have good positive and negative predictive values, which, unlike sensitivity and specificity, are influenced by the prevalence of frailty. In the community, the predictive value of a set of the most common indicators of frailty was found to be very limited once age, sex, and chronic diseases were taken into account [52]. A review of the predictive accuracy of six instruments in diverse settings showed that, because of the low frequency of poor health outcomes, their sensitivity and specificity led to insufficient negative, and even more positive, predictive values [53]. In clinical practice, their predictive value is expected to be lower in primary care than in settings visited by severely diseased patients, such as oncology, or by functionally limited patients, such as those in long-term care. As a consequence, their use by primary care physicians to discuss treatment options with patients should be particularly cautious.

The use of primary care data to measure frailty is attractive, but a recent experience suggested that the frailty index was only moderately able to predict poor outcomes [54, 55]. Other instruments simultaneously applied in primary care showed limited concordance in the detection of frail patients [56–58], and their validation and adjustment to the circumstances of primary care require further research [59–61]. Among the tools tested in primary care to detect frailty, some include socioeconomic dimensions [56, 57, 60, 62]. This approach may be valuable when the purpose is to capture frailty globally in order to reinforce supportive care when needed, including for socioeconomic reasons. However, these instruments may be unethical when the social dimension is predominant in an assessment of frailty used to decide whether a costly intervention is appropriate because disadvantaged individuals will thus be denied care on the grounds of their social vulnerability.

### Screening for frailty in patients with selected conditions

Various frailty indicators are associated with adverse outcomes in patients with coronary heart disease, such as the frailty index, which is strongly and independently related to in-hospital and 1-month mortality in non-ST elevation myocardial infarction [63], or the Edmonton frailty scale, which is associated with adjusted mortality and other outcomes [64]. In patients of the cardiology ward with at least double-vessel coronary artery disease, walking speed better predicted 6-month mortality than the frailty phenotype. In this case, the walking speed had similar positive predictive value and better negative predictive value, but only slightly more than one in ten classified as frail by these two methods saw this outcome [65].

In middle-aged patients with chronic kidney disease not undergoing dialysis, the frailty phenotype prevalence was much higher than that in population-based controls. In addition, Fried’s phenotype was associated with a higher risk of dialysis or death [66].

Airflow limitations and a pulmonary restrictive pattern were found to be cross-sectionally associated with the frailty phenotype in the CHS. An increased risk of developing frailty was observed when they were present at baseline and vice versa. Mortality increased when frailty and respiratory conditions were both present [67].

In oncology, frailty screening has been investigated for its potential to target patients needing referral to comprehensive geriatric assessment (CGA). A review of several instruments suggested an insufficient discriminative power of frailty screening tools to predict impairment. Instruments that had the highest sensitivity had a negative predictive value too low to justify their use for selecting patients for CGA [68]. Others reached the same conclusion [69, 70]. A pilot study suggested a prospective association between grip strength and oncologic treatment toxicity but did not provide information on its predictive ability [71]. The same study failed to show a significant relationship between frailty markers and visits to general practitioners or hospital admissions [72].

### Screening for frailty in the hospital

The frailty index method is attractive for the evaluation of inpatients because a large number of health variables are routinely collected at hospital admission. Several frailty screening tools, including both the frailty index and the frailty phenotype, predicted adverse outcomes at discharge from a geriatric unit and 6 months later, but only the frailty index seemed to have an adequate discriminatory power at both times [73]. Fried’s frailty phenotype was also singled out as a significant risk factor for 6-month adjusted mortality but was not associated with in-hospital falls and delirium [74]. In another study, five different frailty scales were all associated with mortality, readmission, functional decline and a composite outcome but their predictive properties were poor, leading the authors to conclude that frailty scales alone are insufficient to stratify older patients discharged from acute medical units [75].

Associations with adverse outcomes and the accuracy of prediction of frailty indicators were also investigated in specific hospital departments. In trauma patients and emergency departments, the frailty index was strongly related to death and discharge dispositions but not repeat visits to the emergency room [76, 77]. It may, however, be difficult in this setting to measure physical performance to determine the level of Fried’s frailty phenotype; self-assessed weakness and slowness in emergency room patients have been found to be poorly sensitive for this indicator [78].

### Screening for frailty in surgical patients

Most instruments designed to estimate perioperative risk focus on one single organ whereas older patients accumulate multiple diseases. Frailty screening tools might help anesthetists and surgeons to appreciate objectively the global health of their patients [50, 79]. A frailty index was found to be associated with complications and the adjusted mortality risk in emergency general surgery [80] and the frailty phenotype with the complications, length of stay, and discharge dispositions in elective surgery, increasing significantly the predictive power of three perioperative risk indexes [81, 82]. Conflicting results were found in abdominal surgery: an approximation of frailty by routine preoperative data predicted 30-day mortality, and to a lesser extent major morbidity, after lower gastrointestinal surgery in one study [83] while the frailty index was not associated with 30-day postoperative complications in another [84]. In elective non-cardiac, mostly orthopedic surgery, the Edmonton frailty scale was associated with the same outcomes independent of age [85]. Compared to the patients’ and surgeons’ appreciation of frailty, the measured frailty phenotype may correct for unrealistic expectations from patients and an overreliance by clinicians on chronological age [86]. Recent reviews confirmed the relationships between frailty assessments and perioperative outcomes regardless of surgical populations and tools. They also underlined the heterogeneity of research methods and the need for further research to address which assessment method is most predictive [87–89]. A similar conclusion was reached in another review looking at the effectiveness of preoperative CGA on surgery outcomes, despite results predominantly in favor of this type of evaluation [90].

Cardiac surgery is probably the most investigated specialty in research on frailty screening. Walking speed and a score of disability were found to be predictive of mortality or major morbidity in bypass or valve surgery, and both frailty and disability significantly improved the prediction from a cardiac surgery risk score [91]. The Fried phenotype predicts death as well as death or myocardial infarction after percutaneous revascularization [92]. Several reviews have pointed to frailty as an independent risk factor for perioperative morbidity [93] and death [94] in cardiac surgery, with possibly a better predictive ability in older than in younger patients [95]. Improvements in the prediction seem to be modest [94]; thus, as Afilalo et al. emphasized, a positive frailty assessment should not guide rationing decisions but rather designate patients needing reinforced care [96].

### Frailty as a target of individual preventive interventions

Following early calls to design randomized control trials for preventive interventions targeting frailty [97], several projects aimed at building the evidence for action. Overall, physical exercise, with or without nutrition supplements, is the most frequent component of interventions targeting frail older persons [98], and it seems to be beneficial to both physical performance and functional status although the most effective type of exercise remains to be determined [99–102]. Interventions in the community or in primary care settings often share a three-step structure: frailty screening to identify pre-frail or frail older persons, CGA to define individual needs, and a multidimensional intervention to match these needs in the frame of individual care plans [103–115]. The components of interventions include physical exercise, nutrition, lifestyles, cognitive training, medication review, and specific clinical targets and evidence-based care plans for geriatric conditions. Other studies have targeted patients after major abdominal surgery [116], tested limited interventions such as exercise and nutrition [117–119], or relied on nursing home visits [120] or on recommendations to primary care physicians and health and social services [121]. Nevertheless, the evidence regarding the effectiveness of individual interventions designed to prevent an evolution towards more severe stages of frailty and their consequences remains weak because several trials are still in the design or pilot phases.

## Conclusion

Frailty remains a focus of recent research and requires additional investigation in all aspects. Despite our limited knowledge, demographic circumstances raise urgent questions both for public health practitioners and clinicians, explaining the fast diffusion of the frailty concept. Accordingly, several recent guidelines and consensus conferences converged to recommend routine screening for frailty in older adults [2, 47, 122, 123].

Learning by doing will probably be the rule in the next decade. Frailty, as an integrative indicator of health in the whole range of old age, is worth screening to monitor population health. Its effect and inter-relationships with socioeconomic or environmental factors, as determinants of adverse health outcomes, need further research; expected results will help to adapt public policies. Likewise, frailty as a marker of a possibly reversible vulnerability to adverse outcomes is worth screening in clinical settings in order to detect patients in need of CGA. Health and social problems identified from this process may benefit from evidence-based interventions that are not specific to the management of a still obscure frailty syndrome.

In clinical settings, screening for frailty seems particularly appropriate in the absence of functional difficulty. It may however not be useful in already disabled patients because they should systematically benefit from periodic CGA. Although frailty screening is justified—despite a limited predictive accuracy—in prompting geriatric management, making bedside rationing decisions on this basis must be rejected because currently available frailty assessment tools have large false-positive rates.

Continuing research is needed not only to better understand the nature of frailty but also to improve screening tools and test the effectiveness of interventions. Such investigations should accompany rather than delay all of the necessary efforts to respond to needs at the population and individual levels.

## Abbreviations

ADL: Activities of Daily Living
CHS: Cardiovascular Health Study
CGA: Comprehensive Geriatric Assessment
SHARE: Survey of Health, Aging and Retirement in Europe
